# Supplementary material for: Modeling glioblastoma heterogeneity as a dynamic network of cell states
Source: Mol Syst Biol. 2021 Sep 16;17(9):e10105. doi: 10.15252/msb.202010105 (PMC8444284; doi:10.15252/msb.202010105)
Supplement: Supplementary file 5 — Source Data for Figure 3 [file MSB-17-e10105-s001.zip › Figure3A_sourcedata/GSEA_3065/nosigdb_state3.GseaPreranked.1622718426028/GAROFANO_IAVARONE_MTC_SUBTYPE.html]

Details for gene set GAROFANO\_IAVARONE\_MTC\_SUBTYPE[GSEA]

|  || Dataset | state3 |
| Phenotype | NoPhenotypeAvailable |
| Upregulated in class | na\_neg |
| GeneSet | GAROFANO\_IAVARONE\_MTC\_SUBTYPE |
| Enrichment Score (ES) | -0.37544116 |
| Normalized Enrichment Score (NES) | -1.4706305 |
| Nominal p-value | 0.0040431265 |
| FDR q-value | 0.061088417 |
| FWER p-Value | 0.898 |
Table: GSEA Results Summary

  

Fig 1: Enrichment plot: GAROFANO\_IAVARONE\_MTC\_SUBTYPE      
 Profile of the Running ES Score & Positions of GeneSet Members on the Rank Ordered List

  

| PROBE | GENE SYMBOL | GENE\_TITLE | RANK IN GENE LIST | RANK METRIC SCORE | RUNNING ES | CORE ENRICHMENT || 1 | PLD5 |  |  | 97 | 0.348 | 0.0046 | No |
| 2 | PRCP |  |  | 166 | 0.303 | 0.0105 | No |
| 3 | TMEM106B |  |  | 199 | 0.284 | 0.0197 | No |
| 4 | DDRGK1 |  |  | 248 | 0.264 | 0.0262 | No |
| 5 | AIG1 |  |  | 393 | 0.219 | 0.0193 | No |
| 6 | NUDT9 |  |  | 400 | 0.219 | 0.0286 | No |
| 7 | REEP5 |  |  | 429 | 0.213 | 0.0351 | No |
| 8 | DDR1 |  |  | 454 | 0.208 | 0.0418 | No |
| 9 | NDFIP1 |  |  | 464 | 0.206 | 0.0502 | No |
| 10 | PJA2 |  |  | 465 | 0.205 | 0.0596 | No |
| 11 | SLC4A4 |  |  | 555 | 0.192 | 0.0579 | No |
| 12 | PTPRA |  |  | 571 | 0.189 | 0.0648 | No |
| 13 | TMED1 |  |  | 582 | 0.189 | 0.0723 | No |
| 14 | TMEM68 |  |  | 755 | 0.163 | 0.0596 | No |
| 15 | KCNF1 |  |  | 784 | 0.159 | 0.0636 | No |
| 16 | PIGU |  |  | 913 | 0.147 | 0.0554 | No |
| 17 | CLU |  |  | 988 | 0.139 | 0.0531 | No |
| 18 | JAG1 |  |  | 1011 | 0.138 | 0.0568 | No |
| 19 | MLC1 |  |  | 1033 | 0.136 | 0.0605 | No |
| 20 | FJX1 |  |  | 1071 | 0.133 | 0.0623 | No |
| 21 | PLTP |  |  | 1118 | 0.128 | 0.0628 | No |
| 22 | DNAJC18 |  |  | 1212 | 0.121 | 0.0574 | No |
| 23 | CSNK2A1 |  |  | 1252 | 0.118 | 0.0582 | No |
| 24 | FZD3 |  |  | 1258 | 0.117 | 0.0630 | No |
| 25 | CCND2 |  |  | 1286 | 0.116 | 0.0652 | No |
| 26 | ZNF606 |  |  | 1472 | 0.105 | 0.0483 | No |
| 27 | IER5 |  |  | 1491 | 0.103 | 0.0509 | No |
| 28 | STMN3 |  |  | 1523 | 0.102 | 0.0519 | No |
| 29 | TMEM131 |  |  | 1565 | 0.100 | 0.0517 | No |
| 30 | C19orf25 |  |  | 1622 | 0.096 | 0.0495 | No |
| 31 | CDS2 |  |  | 1794 | 0.087 | 0.0335 | No |
| 32 | KIF21A |  |  | 1855 | 0.084 | 0.0303 | No |
| 33 | ZNF721 |  |  | 1879 | 0.082 | 0.0314 | No |
| 34 | MANEAL |  |  | 1896 | 0.081 | 0.0332 | No |
| 35 | SLC12A4 |  |  | 2038 | 0.074 | 0.0201 | No |
| 36 | TMEM59L |  |  | 2066 | 0.073 | 0.0203 | No |
| 37 | ASCC3 |  |  | 2067 | 0.073 | 0.0236 | No |
| 38 | ZNF790 |  |  | 2106 | 0.072 | 0.0225 | No |
| 39 | ZNF181 |  |  | 2127 | 0.071 | 0.0234 | No |
| 40 | ZNF24 |  |  | 2134 | 0.071 | 0.0259 | No |
| 41 | UPF1 |  |  | 2199 | 0.069 | 0.0216 | No |
| 42 | CHERP |  |  | 2219 | 0.067 | 0.0224 | No |
| 43 | ZNF441 |  |  | 2366 | 0.062 | 0.0082 | No |
| 44 | EGFR |  |  | 2416 | 0.060 | 0.0052 | No |
| 45 | FBXO22 |  |  | 2491 | 0.057 | -0.0009 | No |
| 46 | SEMA6D |  |  | 2534 | 0.056 | -0.0032 | No |
| 47 | METTL8 |  |  | 2568 | 0.055 | -0.0046 | No |
| 48 | PGGT1B |  |  | 2590 | 0.054 | -0.0046 | No |
| 49 | STK33 |  |  | 2674 | 0.051 | -0.0120 | No |
| 50 | GAN |  |  | 2727 | 0.049 | -0.0158 | No |
| 51 | TSPAN3 |  |  | 2828 | 0.046 | -0.0254 | No |
| 52 | HEPACAM |  |  | 2847 | 0.046 | -0.0254 | No |
| 53 | DCTN4 |  |  | 2906 | 0.044 | -0.0302 | No |
| 54 | LARS2 |  |  | 2935 | 0.043 | -0.0315 | No |
| 55 | G6PC3 |  |  | 2997 | 0.041 | -0.0368 | No |
| 56 | ZNF44 |  |  | 3029 | 0.040 | -0.0385 | No |
| 57 | ZNF677 |  |  | 3069 | 0.039 | -0.0413 | No |
| 58 | PCBD2 |  |  | 3173 | 0.036 | -0.0517 | No |
| 59 | DIS3L |  |  | 3204 | 0.036 | -0.0536 | No |
| 60 | TMEM205 |  |  | 3288 | 0.033 | -0.0618 | No |
| 61 | PSEN2 |  |  | 3299 | 0.033 | -0.0614 | No |
| 62 | ABHD6 |  |  | 3334 | 0.032 | -0.0639 | No |
| 63 | ZNF134 |  |  | 3373 | 0.031 | -0.0669 | No |
| 64 | ZNF567 |  |  | 3424 | 0.030 | -0.0714 | No |
| 65 | COMMD10 |  |  | 3428 | 0.030 | -0.0704 | No |
| 66 | FEM1A |  |  | 3434 | 0.030 | -0.0696 | No |
| 67 | ZNF350 |  |  | 3439 | 0.029 | -0.0688 | No |
| 68 | PCCB |  |  | 3542 | 0.027 | -0.0795 | No |
| 69 | PFKM |  |  | 3564 | 0.026 | -0.0807 | No |
| 70 | C18orf32 |  |  | 3599 | 0.026 | -0.0835 | No |
| 71 | MOAP1 |  |  | 3639 | 0.025 | -0.0870 | No |
| 72 | KLHL4 |  |  | 3658 | 0.024 | -0.0880 | No |
| 73 | OSBPL6 |  |  | 3700 | 0.023 | -0.0917 | No |
| 74 | CMTM4 |  |  | 3733 | 0.022 | -0.0945 | No |
| 75 | PIGH |  |  | 3750 | 0.021 | -0.0954 | No |
| 76 | ZNF426 |  |  | 3794 | 0.020 | -0.0995 | No |
| 77 | CXCL16 |  |  | 3810 | 0.020 | -0.1003 | No |
| 78 | CHCHD6 |  |  | 3815 | 0.020 | -0.0999 | No |
| 79 | COX18 |  |  | 3854 | 0.019 | -0.1035 | No |
| 80 | FBXL17 |  |  | 3982 | 0.016 | -0.1176 | No |
| 81 | RPRD1A |  |  | 4046 | 0.014 | -0.1244 | No |
| 82 | BTG2 |  |  | 4070 | 0.013 | -0.1265 | No |
| 83 | SCHIP1 |  |  | 4109 | 0.012 | -0.1304 | No |
| 84 | ZNF626 |  |  | 4161 | 0.011 | -0.1358 | No |
| 85 | DTNA |  |  | 4203 | 0.009 | -0.1402 | No |
| 86 | THAP2 |  |  | 4257 | 0.008 | -0.1460 | No |
| 87 | PDCD2L |  |  | 4267 | 0.008 | -0.1467 | No |
| 88 | GEMIN6 |  |  | 4316 | 0.006 | -0.1521 | No |
| 89 | BCDIN3D |  |  | 4395 | 0.004 | -0.1610 | No |
| 90 | AHCY |  |  | 4459 | 0.002 | -0.1683 | No |
| 91 | BAG5 |  |  | 4468 | 0.002 | -0.1691 | No |
| 92 | ZNF844 |  |  | 4476 | 0.002 | -0.1698 | No |
| 93 | PRMT3 |  |  | 4529 | 0.001 | -0.1759 | No |
| 94 | ZBTB22 |  |  | 4546 | -0.000 | -0.1778 | No |
| 95 | MED9 |  |  | 4585 | -0.001 | -0.1822 | No |
| 96 | ZCCHC10 |  |  | 4619 | -0.002 | -0.1860 | No |
| 97 | NIPAL3 |  |  | 4687 | -0.003 | -0.1936 | No |
| 98 | ANAPC10 |  |  | 4696 | -0.004 | -0.1944 | No |
| 99 | TTC23 |  |  | 4750 | -0.005 | -0.2004 | No |
| 100 | FRS2 |  |  | 4756 | -0.005 | -0.2008 | No |
| 101 | LFNG |  |  | 4861 | -0.007 | -0.2126 | No |
| 102 | TMEM199 |  |  | 4870 | -0.008 | -0.2132 | No |
| 103 | ZNF84 |  |  | 4883 | -0.008 | -0.2142 | No |
| 104 | ELMO2 |  |  | 4955 | -0.010 | -0.2221 | No |
| 105 | VPS16 |  |  | 4990 | -0.011 | -0.2256 | No |
| 106 | PLEKHF2 |  |  | 4995 | -0.011 | -0.2255 | No |
| 107 | SYT17 |  |  | 5035 | -0.012 | -0.2295 | No |
| 108 | SOX21 |  |  | 5077 | -0.013 | -0.2338 | No |
| 109 | NDUFS7 |  |  | 5079 | -0.013 | -0.2333 | No |
| 110 | SLC25A26 |  |  | 5090 | -0.013 | -0.2338 | No |
| 111 | HKR1 |  |  | 5112 | -0.014 | -0.2357 | No |
| 112 | BNIP1 |  |  | 5151 | -0.014 | -0.2395 | No |
| 113 | C18orf25 |  |  | 5181 | -0.015 | -0.2422 | No |
| 114 | HINT3 |  |  | 5260 | -0.017 | -0.2505 | No |
| 115 | CA2 |  |  | 5274 | -0.018 | -0.2512 | No |
| 116 | PREX1 |  |  | 5327 | -0.019 | -0.2564 | No |
| 117 | ZNF576 |  |  | 5362 | -0.020 | -0.2595 | No |
| 118 | ZNF561 |  |  | 5366 | -0.020 | -0.2589 | No |
| 119 | TRIM4 |  |  | 5415 | -0.021 | -0.2636 | No |
| 120 | STAU2 |  |  | 5428 | -0.022 | -0.2640 | No |
| 121 | ZNF317 |  |  | 5433 | -0.022 | -0.2635 | No |
| 122 | TRIM23 |  |  | 5459 | -0.023 | -0.2653 | No |
| 123 | NFS1 |  |  | 5499 | -0.024 | -0.2688 | No |
| 124 | TFPT |  |  | 5508 | -0.024 | -0.2686 | No |
| 125 | WDR20 |  |  | 5591 | -0.027 | -0.2770 | No |
| 126 | DNAL1 |  |  | 5599 | -0.027 | -0.2766 | No |
| 127 | BAIAP2 |  |  | 5790 | -0.032 | -0.2973 | No |
| 128 | MAP2K5 |  |  | 5811 | -0.033 | -0.2981 | No |
| 129 | RCHY1 |  |  | 5907 | -0.036 | -0.3076 | No |
| 130 | SDHAF1 |  |  | 6002 | -0.040 | -0.3168 | No |
| 131 | C12orf73 |  |  | 6005 | -0.040 | -0.3152 | No |
| 132 | SLC27A5 |  |  | 6028 | -0.040 | -0.3159 | No |
| 133 | SH3PXD2B |  |  | 6049 | -0.041 | -0.3164 | No |
| 134 | ALDH6A1 |  |  | 6109 | -0.043 | -0.3213 | No |
| 135 | DHRSX |  |  | 6125 | -0.044 | -0.3211 | No |
| 136 | TIMM9 |  |  | 6180 | -0.046 | -0.3253 | No |
| 137 | CCDC102B |  |  | 6213 | -0.047 | -0.3269 | No |
| 138 | TMEM147 |  |  | 6237 | -0.048 | -0.3274 | No |
| 139 | THG1L |  |  | 6378 | -0.052 | -0.3414 | No |
| 140 | ZNF414 |  |  | 6400 | -0.053 | -0.3415 | No |
| 141 | FDXR |  |  | 6438 | -0.054 | -0.3433 | No |
| 142 | SUCLG2 |  |  | 6466 | -0.055 | -0.3440 | No |
| 143 | FARS2 |  |  | 6495 | -0.056 | -0.3447 | No |
| 144 | PPP2R2B |  |  | 6515 | -0.057 | -0.3443 | No |
| 145 | RAB39B |  |  | 6681 | -0.065 | -0.3607 | No |
| 146 | ENOPH1 |  |  | 6746 | -0.068 | -0.3650 | No |
| 147 | PCIF1 |  |  | 6747 | -0.068 | -0.3619 | No |
| 148 | BTBD2 |  |  | 6755 | -0.069 | -0.3596 | No |
| 149 | C1orf53 |  |  | 6768 | -0.069 | -0.3578 | No |
| 150 | NDUFS3 |  |  | 6821 | -0.072 | -0.3606 | No |
| 151 | ACYP2 |  |  | 6848 | -0.073 | -0.3603 | No |
| 152 | MRPL46 |  |  | 6904 | -0.076 | -0.3633 | No |
| 153 | ISCA2 |  |  | 6940 | -0.078 | -0.3638 | No |
| 154 | NDUFS4 |  |  | 6944 | -0.078 | -0.3606 | No |
| 155 | MESP1 |  |  | 6967 | -0.079 | -0.3595 | No |
| 156 | CAMK2D |  |  | 6988 | -0.080 | -0.3582 | No |
| 157 | ECSIT |  |  | 7003 | -0.081 | -0.3562 | No |
| 158 | MTHFS |  |  | 7053 | -0.084 | -0.3581 | No |
| 159 | KCTD6 |  |  | 7072 | -0.085 | -0.3563 | No |
| 160 | CRLS1 |  |  | 7078 | -0.086 | -0.3529 | No |
| 161 | IDH3B |  |  | 7099 | -0.087 | -0.3513 | No |
| 162 | ALDH3A2 |  |  | 7250 | -0.097 | -0.3644 | No |
| 163 | RAD51C |  |  | 7264 | -0.098 | -0.3615 | No |
| 164 | DIRAS1 |  |  | 7267 | -0.098 | -0.3572 | No |
| 165 | MAPK8IP1 |  |  | 7274 | -0.099 | -0.3534 | No |
| 166 | NUDT16 |  |  | 7277 | -0.099 | -0.3491 | No |
| 167 | IRS2 |  |  | 7285 | -0.099 | -0.3454 | No |
| 168 | CINP |  |  | 7335 | -0.103 | -0.3464 | No |
| 169 | RNF141 |  |  | 7403 | -0.108 | -0.3493 | No |
| 170 | RAB9A |  |  | 7606 | -0.128 | -0.3671 | No |
| 171 | TSC22D4 |  |  | 7610 | -0.129 | -0.3616 | No |
| 172 | CKB |  |  | 7659 | -0.134 | -0.3610 | No |
| 173 | PTPMT1 |  |  | 7669 | -0.135 | -0.3559 | No |
| 174 | CMBL |  |  | 7837 | -0.156 | -0.3683 | Yes |
| 175 | ZYX |  |  | 7853 | -0.158 | -0.3628 | Yes |
| 176 | AMZ2 |  |  | 7936 | -0.174 | -0.3644 | Yes |
| 177 | UBE2F |  |  | 7937 | -0.174 | -0.3565 | Yes |
| 178 | CHCHD5 |  |  | 7939 | -0.175 | -0.3486 | Yes |
| 179 | NUDT16L1 |  |  | 7949 | -0.177 | -0.3416 | Yes |
| 180 | ATP6V0E2 |  |  | 8025 | -0.189 | -0.3417 | Yes |
| 181 | BPGM |  |  | 8071 | -0.197 | -0.3379 | Yes |
| 182 | ELOF1 |  |  | 8109 | -0.205 | -0.3329 | Yes |
| 183 | UQCRB |  |  | 8141 | -0.214 | -0.3267 | Yes |
| 184 | JUND |  |  | 8150 | -0.214 | -0.3179 | Yes |
| 185 | C20orf27 |  |  | 8155 | -0.216 | -0.3084 | Yes |
| 186 | NLRP1 |  |  | 8171 | -0.220 | -0.3001 | Yes |
| 187 | MRPS26 |  |  | 8177 | -0.221 | -0.2906 | Yes |
| 188 | GAMT |  |  | 8235 | -0.236 | -0.2865 | Yes |
| 189 | MRPL54 |  |  | 8301 | -0.258 | -0.2823 | Yes |
| 190 | UQCRFS1 |  |  | 8310 | -0.260 | -0.2713 | Yes |
| 191 | TRIAP1 |  |  | 8318 | -0.262 | -0.2601 | Yes |
| 192 | PFDN5 |  |  | 8373 | -0.282 | -0.2536 | Yes |
| 193 | SRI |  |  | 8395 | -0.292 | -0.2427 | Yes |
| 194 | CYCS |  |  | 8405 | -0.297 | -0.2301 | Yes |
| 195 | TIMM13 |  |  | 8438 | -0.309 | -0.2197 | Yes |
| 196 | BLOC1S1 |  |  | 8483 | -0.331 | -0.2097 | Yes |
| 197 | RPS27L |  |  | 8528 | -0.351 | -0.1988 | Yes |
| 198 | PHPT1 |  |  | 8596 | -0.398 | -0.1884 | Yes |
| 199 | HIGD1A |  |  | 8616 | -0.419 | -0.1715 | Yes |
| 200 | COX6B1 |  |  | 8631 | -0.435 | -0.1532 | Yes |
| 201 | POLR2I |  |  | 8643 | -0.447 | -0.1341 | Yes |
| 202 | HIGD2A |  |  | 8654 | -0.458 | -0.1143 | Yes |
| 203 | CDKN1A |  |  | 8657 | -0.461 | -0.0935 | Yes |
| 204 | TOMM7 |  |  | 8666 | -0.470 | -0.0729 | Yes |
| 205 | H3F3B |  |  | 8687 | -0.504 | -0.0522 | Yes |
| 206 | RPS15 |  |  | 8723 | -0.582 | -0.0297 | Yes |
| 207 | RPL41 |  |  | 8743 | -0.718 | 0.0009 | Yes |
Table: GSEA details [plain text format]

  

Fig 2: GAROFANO\_IAVARONE\_MTC\_SUBTYPE: Random ES distribution      
 Gene set null distribution of ES for **GAROFANO\_IAVARONE\_MTC\_SUBTYPE**
